# Heat capacity signature of frustrated trimerons in magnetite

**DOI:** 10.1038/s41598-020-67955-x

**Published:** 2020-07-02

**Authors:** S. Sahling, J. E. Lorenzo, G. Remenyi, C. Marin, V. L. Katkov, V. A. Osipov

**Affiliations:** 1 Institut für Festkörper- und Materialphysik, Technische Universität Dresden, 01069 Dresden, Germany; 2 Institut Néel, Université Grenoble Alpes - CNRS:UPR2940, 38042 Grenoble, France; 3 Université Grenoble Alpes, CEA, IRIG-Pheliqs, 38000 Grenoble, France; 4 Bogoliubov Laboratory of Theoretical Physics, Joint Institute for Nuclear Research, 141980 Dubna, Moscow region Russia

**Keywords:** Ferromagnetism, Electronic properties and materials, Ferroelectrics and multiferroics

## Abstract

Recently it has been proposed that the long-range electronic order formed by trimerons in magnetite should be frustrated due to the great degeneracy of arrangements linking trimerons. This result has important consequences as charge ordering from the condensed minority band electrons leads to a complex 3D antiferro orbital order pattern. Further more, the corner sharing tetrahedra structure of spinel B-sites supports frustration for antiferromagnetic alignments. Therefore frustration due to competing interactions will itself induce disorder and very likely frustration in the spin orientations. Here we present very low temperature specific heat data that show two deviations to the magnons and phonons contributions, that we analyze in terms of Schottky-type anomalies. The first one is associated with the thermal activation across both ferroelastic twin and ferromagnetic anti-phase domains. The second Schottky-type anomaly displays an inverse (1/H) field dependence which is a direct indication of the disordered glassy network with macroscopically degenerated singular ground states.

## Introduction

Magnetite in the high temperature phase ($T > 130\,\text {K}$) is a classical ferrimagnet with a Néel temperature $T_N = 850\,\text {K}$^1,2^. The cubic lattice (space group $\text {Fd}\bar{3}\text {m}$) has an inverse $\text {AB}_2\text {O}_4$ spinel structure $(\text {Fe}^{3+}[\text {Fe}^{3+}\text {Fe}^{2+}]\text {O}_4)$, where $\text {Fe}^{3+}$ occupies the tetrahedrally coordinated A-sites with a magnetic moment of $5\,\mu _B$. $\text {Fe}^{3+}$ ($3\text {d}^5$) and $\text {Fe}^{2+}$ ($3\text {d}^6$) in the ratio 1:1 are the octahedrally coordinated B-sites with the magnetic moments $5 \mu _B$ and $4 \mu _B$, respectively. The total magnetic moment of magnetite is $4 \mu _B$ per formula unit since an antiferromagnetic coupling exists between the A and B Fe-sites^2^. The magnetization easy axis is aligned along the (111) direction and four types of micron size ferromagnetic domains, each one of them along the 4 different directions, are present below $T_N$.

The understanding of the metal-semiconducting transition occurring at around $T_V\approx 123\,\text {K}$ (the Verwey transition), where the electrical conductivity drops by more than 2 orders of magnitude, along with anomalies in basically all experimental data^3^, is still a subject of active interest. As a result of this phase transition the cubic lattice, $a_c$, transforms into a monoclinic one ($\text {Fd}\bar{3}\text {m} \rightarrow \text {Cc}$) and the unit cell becomes 4 times larger ($\sqrt{2} a_c \times \sqrt{2} a_c \times 2a_c$). Initially it was considered that a bimodal arrangement of charges in the form of $\text {Fe}^{2.5+\delta }\text {-Fe}^{2.5-\delta }$ ions sets in as a result of the phase transition^4,5^. The charge ordering (CO) developing below $T_V$ turns out to be more complex than initially thought and further studies have concluded that the actual CO is in the form of “trimerons”^6^, with charge disproportionation amounting to 0.2 electrons among the B-sites.

The symmetry lost at this transition involves the formation of up to 24 ferroelastic twin domains that are going to reduce the magnetic domain size and affect its distribution in unsought and complex ways. Medrano et al. have investigated the twin domains in the low-temperature phase of magnetite from synchrotron-radiation X-ray topographs^7^ unraveling the monoclinic domain structure is sensitive to a magnetic field. Recent electron holography and Lorentz microscopy experiments^8,9^ have revealed a very complex network of interactions between the magnetic domains and the ferroelastic twin domains below $T_V$, thus confirming previous results on a strong influence on the low temperature properties of magnetite.

Magnetic after effect^3,10^, ac-magnetic susceptibility^11–13^, and dielectric permitivity^14,15^ experiments have shown the occurrence of glassy-like states below about 50 K. All these susceptibilities exhibit strong frequency dependencies obeying Arrhenius activation laws, albeit of different attempt frequencies and activation energies. Specific heat experiments have reported a broad deviation to the Debye law with a maximum in the $C_P/T^3$ plot at 35 K. This is reminiscent of a similar anomaly detected in glasses and incommensurate systems but at much lower temperatures and known as the boson peak. Whether this is related to the glassy behavior or it is a coincidental deviation of the low temperature phonon behavior is not clear yet. No thermodynamic phase transition has been detected below $T_V$. These numerous studies have concluded that up to 2 different effects are at the origin of this glass-like dynamics: freezing out of domain wall motion followed at lower temperature by a quantum-mechanically tunneling. Finally ac-magnetic susceptibility experiments in magnetite nanoparticles^16^ have revealed an identical behavior as those observed in single crystal, thin films and powdered samples^11–13^ which hints to the possibility that this glassy behavior (both dielectric relaxor and spin glass) is intrinsic to the magnetite low temperature ground state. A recent study^17^ has proposed that the long-range electronic order formed by trimerons should be frustrated due to the great degeneracy of arrangements linking trimerons.

The temperature and magnetic field dependence of the heat capacity is a powerful tool to provide fundamental information on various thermodynamic subsystems in solids. We have used this technique to unravel deviations to the phonon temperature dependence at very low temperatures in incommensurate charge density waves compounds^18,19^ that display dielectric glassy behaviors^18^ very similar to those found in magnetite^14,15^. Following this analogy we have revisited the heat capacity in magnetite from room temperature down to 50 mK, paying special attention to the very low temperatures. Indeed, specific heat data in the temperature range $0.05 \text { K}< \text {T} < 1 \text { K}$ are lacking. In this region, we found two contributions with a fundamentally different behavior under magnetic field that we associate with two separate thermodynamic subsystems.

## Results

**Figure 1:**
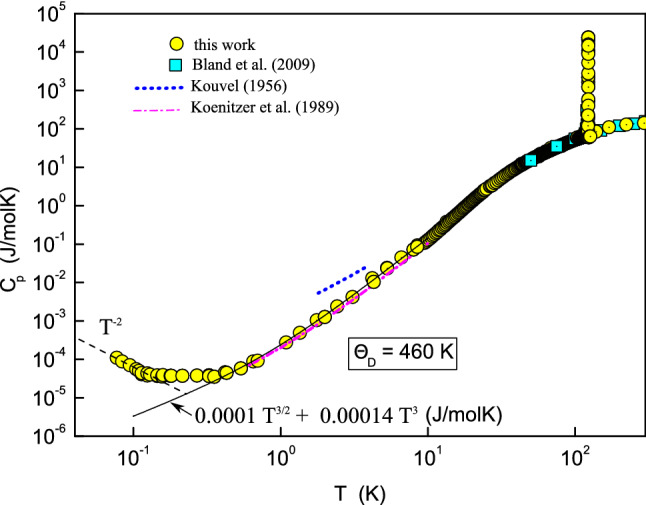
Log-log plot of the equilibrium heat capacity $C_p(T)$ as a function of temperature without magnetic field (together with data from the literature^20–22^). Temperature ranges from 0.05 to 292 K and the measured heat capacity spans 10 orders of magnitude.

**Figure 2:**
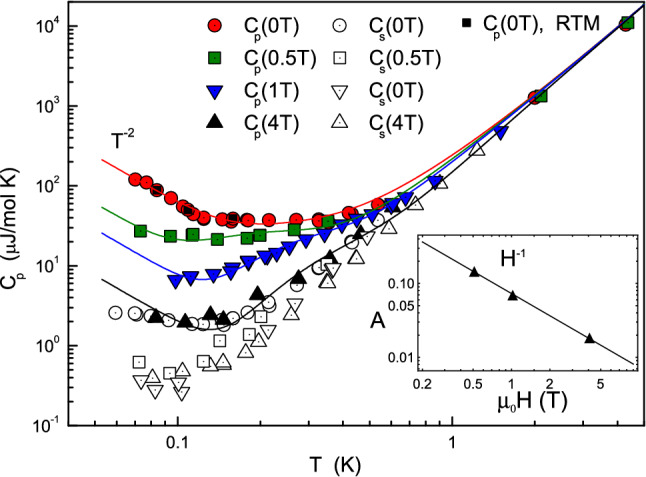
Uncolored (or white) symbols are the short-time heat capacity, $C_s$, measured with the pulse method at 0.1 s whereas colored symbols represent the equilibrium heat capacity, $C_p$, measured by the pulse and relaxation time methods as function of temperature and different magnetic field parallel to the **c**-axis. Black squares represent the result of RTM measurements. Solid lines are calculated by Eq. (1) with parameters given in Table 1. The inset is a log-log plot of the amplitude of the Schottky anomaly versus the applied magnetic field, displaying the 1/H dependence.

Zero field heat capacity is shown in Fig. 1 and the results are in good agreement with previous studies^20–22^ albeit carried out in a narrower temperature window. Previously reported heat capacity experiments down to 300 mK have shown no deviation to the spin wave and phonon contribution^21^. Below $\approx 1\,\text {K}$ the temperature relaxation does not follow a single exponential law (see Supplementary Information). In accordance with previous works^23^ we have defined a short time heat capacity ($C_s$), measured at 0.1 s after the heat pulse, and the equilibrium heat capacity ($C_p$). Both are shown in Fig. 2 as a function of temperature and for three different values of the magnetic field. In addition three points were measured by the relaxation time method (RTM) at 0.084, 0.109 and 0.159 K thus showing that the results of the pulse method and the RTM are identical (See Supplementary Information).

**Table 1:** Results of the fit of data in Figs. 2 and 3 with Eq. (1).

$\mu _0 H$ (T)	*A* ($\upmu \text {J K/mol}$)	$B (\Delta _2)^2$ ($\upmu \text {J K/mol}$)	*B* ($\upmu \text {J K/mol}$)	$\Delta _2$ (K)
0	0.59	13.1	25.3	0.72
0.5	0.151	13.2	45.4	0.54
1	0.072	30.7	40.6	0.87
4	0.019	29.4	20.4	1.2
at 1 T in $T_v$	0.0456	–	–	–

The short-time heat capacity remains constant below 300 mK at zero field (white circles in Fig. 2) and is up to 10 times smaller than its equilibrium value. 90 % of the measured heat capacity is defined by some system with surprisingly slow relaxation.

A small magnetic field reduces both the short-time and the equilibrium heat capacities. $C_s$ already saturates above 0.5 T at values close to those expected from the extrapolation of the phonon contribution measured above 1 K (white squares in Fig. 2). It reflects the fact that the component of the ‘anomalous’ system displaying slow relaxation in the short-time heat capacity is relatively small and is effectively suppressed by the magnetic field. $C_p$ decreases continuously with increasing magnetic field and yet exceeds the short-time heat capacity even at 4 T (black triangles in Fig. 2).

The equilibrium heat capacity grows as $T^{-2}$ on decreasing temperature below 150 mK and in the absence of a magnetic field. In line with our model, it is the tail of the Schottky-type anomaly peaking at even lower temperatures, outside of the window of our experiment.

A plateau in the equilibrium heat capacity appears as evident between 150 and 600 mK. We believe that this plateau originates from a two-level system with relatively narrow distribution that has been commonly observed in glasses, incommensurate systems, etc.^19,23,24^.

Accordingly, we fit the specific heat data using a two-component Schottky contribution (let us call them *A* and *B*) together with the lattice and spin-wave contributions

$$\begin{aligned} C_p(T) = A \left( \frac{1}{T}\right) ^2 + B \left( \frac{\Delta _2}{T}\right) ^2 \frac{e^{\Delta _2/T}}{(1+e^{\Delta _2/T})^2} + C_{ph} + C_{sw}. \end{aligned}$$

*A* is proportional to the density of two-level states of the first type and the energy gap between them, *B* describes the density of two-level states of the second type, and $\Delta _2$ is the energy gap. The estimated parameters giving good agreement with the experiment (see solid lines in Fig. 2) are presented in Table 1.

**Figure 3:**
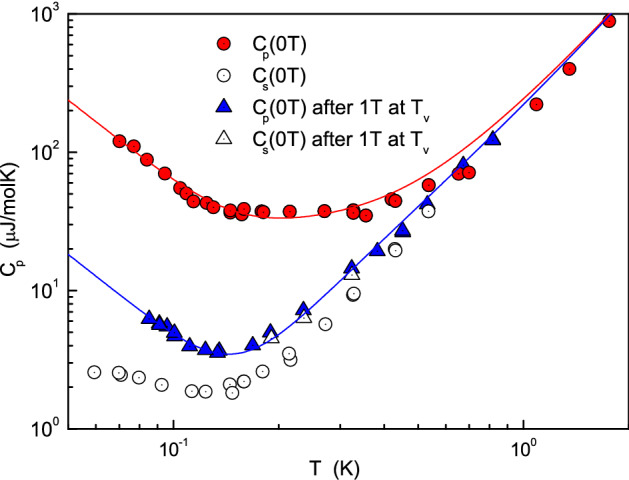
Circles represent the equilibrium $C_p(T)$ (red) and the short time heat capacity $C_s(T)$ (uncolored) measured without magnetic field. Triangles represent the different heat capacities after cooling the sample through the Verwey transition in a magnetic field of 1T and later removed. Lines are the result of the fit with the model in Eq. (1) with parameters given in Table 1.

The application of the magnetic field at the Verwey transition leads to a strong decrease in the heat capacity at temperatures below 0.7 K. Our analysis shows that the model in Eq. (1) perfectly describes the experimental data provided that (i) the second Schottky term (type *B*) completely disappears and (ii) there is a residual magnetic field of 0.37 T (see the blue line in Fig. 3). The residual magnetization indicates the presence of an intrinsic field thus confirming the dominant magnetic single domain formation.

Therefore we conclude that the type-$B$ Schottky term originates from the multidomain magnetic domain wall structure. In the aligned and de-twinned crystal both long (or equilibrium heat capacity) and short time heat capacities are found to be close to each other. This confirms that such slow two-level system is responsible for the anomalous behavior. An additional analysis of the RTM spectra (see Supplementary Information) reveals the thermoactivation nature of the slow process and gives an activation energy of $\approx 0.5 \,\text { K}$, which is close to the values found from the fit by Schottky term *B* (see Table 1).

## Discussion

The application of 0.5T after zero field cooling leads to the disappearance of the excess in the short time heat capacity. Further increasing of the field does not yield a significant change. By X-ray topography experiments, Medrano et al.^7^ have identified this field with a decrease in the number of twin domains and with the axis switching of the domains having their **c**-axis orthogonal to the magnetic field direction.

With regard to the equilibrium heat capacity, the first subsystem is sensitive to the application of a magnetic field. Namely, the corresponding Schottky parameter *A* is inversely related to the magnetic field (see inset in Fig. 2). This inverse dependence is a rather unexpected result and, at first glance, it could be interpreted as a decrease of either a gap or of the number of two-level systems with increasing field. The 1/*H*-like dependence was observed earlier in pyrochlore titanates compounds displaying the spin ice ground state^25^. A very similar feature was found in a wide class of exactly solvable frustrated models based on the idea of highly macroscopically degenerated single-point ground state^26^. The Schottky anomaly is a consequence of a decrease in entropy with a decrease in temperature. At the same time, a decrease in the amplitude of this anomaly with an increase in the magnetic field is associated with an increase in entropy when the field approaches the single point ground state.

The *A* Schottky-like term, which tail is observed in our experiment, is a manifestation of a frustrated magnetic lattice. Indeed, the temperature and magnetic field behavior agree with the general results for the entropy and heat capacity for a wide class of frustrated lattices^26^. Unfortunately, quantitative analysis in the way to determine the type of frustrated lattice (system) is hindered by the fact that (i) only the tail of this anomaly is observed in the experiment, and (ii) the magnetic field strength available turns out to be insufficient to unravel the actual landscape of interactions.

We propose that the frustrated network of trimerons^17^ is at the origin of this *A* contribution. The trimerons CO in the low-temperature magnetite structure may be regarded as orbital molecules behaving as one electron quasiparticles with effective spin-1/2^27^. Charge and orbital fluctuations couple with magnetic order, and the ensuing electronic instabilities induce a certain degree of disorder in the amplitude and direction of the magnetic moments at individual Fe $B$-sites. The occurrence of non-collinear magnetic orbital components at the $B$-sites has been proposed^28–30^ and has been very recently observed by resonant magnetic elastic (and inelastic) scattering experiments^31,32^. This is an important finding as antiferromagnetic orderings in the spinel $B$-site are known to display frustration^33^. How this intrinsic magnetic frustration or that resulting from the trimerons CO or both together affect the low temperature properties of magnetite is still under discussion.

In addition, the propose orbital ordering also supports multiferroicity^30^ which may explain the dielectric susceptibility results, the appearance of an spontaneous polarization and the occurrence of a dipolar glassy state^14,15^. Moreover our results are in good agreement with the overall trend displayed by glassy systems. The dielectric/magnetic susceptibilities exhibiting a wide temperature range of relaxations are followed by a large upturn of the specific heat at very low temperatures and non-Debye relaxations of the heat across the sample.

## Methods

### Sample

The sample used for this experiment is a large, very high quality, synthetic single crystal rod of mass of 5.72545 g coming from a batch of samples that have been used in previous X-ray and neutron diffraction experiments^5,34^.

### Cryostat and cryogenics

The experiment was performed in a $^3\text {He-}^4\text {He}$ dilution refrigerator inside an 11 T magnet. The pulse and relaxation time methods (RTM) were used to extract the specific heat (See Supplementary Information). The stability of our device has been tested against the first order transition (more than 6 hours at this temperature) where we found an increase of the heat capacity by a factor of 400 right at $T_V$. To the best of our knowledge this is the first time that such long time measurements have been reported in magnetite. In addition, the value of $T_V=123.6\,\text {K}$ and sharp spike at $T_V$ warrants the quality and stoichiometry of the sample.

### Application of a magnetic field at the Verwey transition temperature

Cooling the crystal in a magnetic field produced a dominant single domain (see also^6^). Concomitant application of pressure is mandatory to create an actual single domain. But this is unfortunately precluded in our experiment, in view of the large single crystal required by the low temperature measurements. After warming the sample well above $T_V$ a magnetic field of 1 T was applied at 130 K, slightly above $T_V$, and the sample was field cooled down to 0.1 K. The cooling rates through the Verwey transition of the previous experiment and of this one are nearly identical. Then the magnetic field was switched off and, as before, the heat capacity was measured on heating. A detailed description of the measuring technique is given in the Supplementary Information.

### Application of a magnetic field at low temperature

A magnetic field of varying intensity was applied along the long axis of the sample, the $\mathbf{c}$-axis. A magnetic field of 0.5 T was firstly introduced at the lowest attainable temperature, $\approx 0.05$ K, resulting in sample heating up to 0.3 K due to magnetocaloric effect. The sample was cooled back to base temperature and the specific heat was measured from low to high temperatures, typically up to 10K. The sample was cooled again under field down to base temperature and the procedure was repeated for 1 T and 4 T.

### Fitting high temperature specific heat

The heat capacity between 1 and 10 K has been fitted by the expression $L T^{3/2} + \beta T^3$, which corresponds to the contribution of spin-waves $C_{sw}$ and phonons $C_{ph}$, respectively. The value of $\beta = 0.14\,\text {mJ}/(\text {mol K}^4)$ yields a Debye temperature of 460 K ($\Theta _D \simeq (13608/\beta )^{1/3}$). This is among the lowest values of $\Theta _D$ reported for magnetite single crystal up to date. Those cited in the literature are in the range from 480 to 660 K^21,35^. In addition, the derived value of $L = 0.1\,\text {mJ}/(\text {mol K}^{5/2})$ allows us to estimate the magnetic exchange integral for the nearest-neighbor iron ions located in the tetrahedral (A) and octahedral (B) cation sites as $J_{AB} = 2.48\,\text {meV}$ (28.8 K). These values are in excellent agreement with those reported in the literature^21^ for a sample with a nearly-ideal stoichiometry, $L=0.108\,\text {mJ}/(\text {mol K}^{5/2})$ and $J_{AB} = 2.35\,\text {meV}$, which again confirms the good quality of our crystal.

Spin-wave theory accounts for the suppression of the spin-wave contribution in a magnetic field^36,37^. Namely

1 $$\begin{aligned} C_{sw} = (F/F_0) L T^{3/2}, \end{aligned}$$

where

2 $$\begin{aligned} F(H/T) = \frac{1}{4\pi ^2}\int \limits _{\frac{g \mu _B H}{k_B T}}^{\infty } \frac{x^2 e^x}{(e^x -1)^2} \sqrt{x - \frac{g \mu _B H}{k_B T}} dx. \end{aligned}$$

We use here the value $g=2.06$ (below $T_V$) in accordance with the ferromagnetic resonance measurements in magnetite single crystals^38^, $F_0 =F(0) =0.113$.

## Supplementary information

Supplementary file1 (pdf 1,682 kb)
