# Supplementary material for: Heat capacity signature of frustrated trimerons in magnetite
Source: Sci Rep. 2020 Jul 2;10:10909. doi: 10.1038/s41598-020-67955-x (PMC7331697; doi:10.1038/s41598-020-67955-x)
Supplement: Supplementary file 1 — Supplementary file1 (pdf 1,682 kb) [file 41598_2020_67955_MOESM1_ESM.pdf]

Supplementary Information
Heat capacity signature of frustrated trimerons in magnetite

S. Sahling,[1,2] J.E. Lorenzo,[2,*] G. Remenyi,[2] C. Marin,[3] V.L. Katkov,[4,†] and V.A. Osipov[4,‡]

[1]*Institut für Festkörper- und Materialphysik, Technische Universität Dresden, D-01069 Dresden, Germany*
[2]*Institut Néel, Université Grenoble Alpes - CNRS:UPR2940, 38042 Grenoble, France.*
[3]*SPSMS, UMR-E 9001, CEA-INAC/UJF-Grenoble 1, IMAPEC, F-38054 Grenoble, France*
[4]*Bogoliubov Laboratory of Theoretical Physics, Joint Institute for Nuclear Research, 141980 Dubna, Moscow region, Russia*

I. EXPERIMENT.

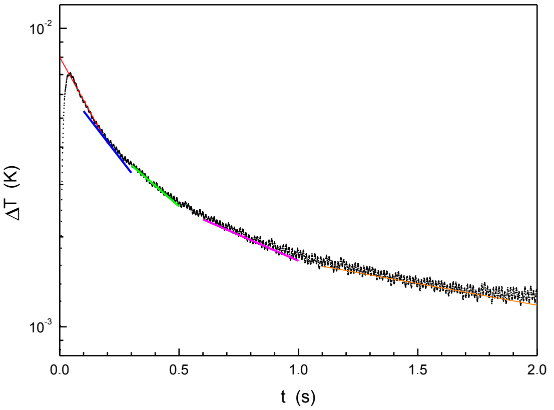

FIG. 1. The temperature difference $\Delta T = T(t) - T_0$ as a function of time at nominal temperature $T_0 = 0.159$ K after a short heat pulse $t_h = 10$ ms. The perturbation introduced by the measurement, $\Delta T_{max}/T_0 \approx 5\%$, is very small. A non exponential law was found for $t < t_{in} = 6s$. A time dependent relaxation time $\tau(t)$ and heat capacity $C_s(t)$ were determined according to Eqs. (6) and (7). From the tangent straight lines shown with different colors, the corresponding parameters at $t = 0.1$ s, 0.2 s, 0.4 s, 0.8 s and 1.6 s were obtained. Finally an exponential law was observed for $t > t_{in}$ only with an equilibrium relaxation time $\tau_{eq} = 18s$. These results are shown in Fig. 2.

The sample used for this experiment is a rod of mass of 5.72545 g coming from a batch of samples that have been used in previous X-ray and neutron diffraction experiments[1,2]. The experiment was performed in a ^{3}He-^{4}He dilution refrigerator inside an 11 T magnet. The pulse and relaxation time methods were used to extract the specific heat. The sample, along with a home-made AuGe thermometer[3] and a Pt-W heater, was attached to the sample holder and wrapped with thin nylon threads. The **c**-axis of the sample was oriented parallel to the magnetic field to within a few degrees. Importantly the time resolution in our experiments is 0.5 ms using a special technique for measuring the resistance of the thermometer. The relaxation time of the AuGe thermometer and the Pt-W heater is less than 0.1 ms even at the lowest temperature equal to 50 mK. An overheating of the thermometer is excluded since the heater and the thermometer were attached to opposite ends of the sample like a sandwich. Thus we determine the lowest limit for the relaxation to about 1 ms.

In the following we discuss the measuring technique paying special attention to the very low temperature data, albeit these methods are used at higher temperatures as well. In the pulse method, the temperature starts to relax after a short heat pulse ($t_h = 10$ ms) as it can be seen in Fig. 1. Below 1K the first stages of the relaxation do not follow an exponential behavior and it only becomes exponential after a time period t_{in}. We extrapolate it back to the time moment $t_h/2$:

$$\Delta T(t_h/2) = \Delta T_0 \exp(-t_h/2\tau). \quad (1)$$

The heat capacity is obtained as

$$C_p = \Delta Q/\Delta T(t_h/2). \quad (2)$$

The amount of heat can be calculated using Joule's law

$$\Delta Q = U_h I_h t_h, \quad (3)$$

where U_h and I_h are the heater voltage and current, respectively. The heat link R_{hl} between the sample and sample holder can be determined from

$$R_{hl} = \tau/C_p. \quad (4)$$

It can be proofed by a separate measurement with a permanent heating power:

$$R_p = \frac{I_h U_h}{\Delta T}. \quad (5)$$

R_p equals R_{hl} in the absence of an inner relaxation process. Otherwise we find $R_{hl} < R_p$.

A non-exponential dependence of the temperature difference $\Delta T = T(t) - T_0$ is obtained at short time ($t < t_{in}$, where t_{in} corresponds to the maximum relaxation time of the inner relaxation time spectrum), if an inner relaxation process exists in the sample. In this case, we can fit the data close to a given time t' by an exponential law

$$\Delta T(t, t') = \Delta T_0(t') \exp[-t/\tau(t')] \quad (6)$$

(see Fig. 1) and introduce a time dependent heat capacity

$$C_s(t) = \Delta Q/\Delta T(t_h/2, t). \quad (7)$$

In this way we determine the part of the heat capacity which is in quasi-equilibrium with phonons at a given

time. $C_s(t)$ equals the equilibrium value C_p if the measuring time is longer than t_{in}, i.e., for $t > t_{in}$ the heat capacity is constant (see Fig. 2). Thus, the ratio $C_s(t)/C_p$ can be used to determine t_{in}. This parameter can be obtained from the time dependence of τ_s (see Fig. 2) that also becomes constant for $t > t_{in}$.

Notice that the observed non-exponential time dependence of the temperature after the heat pulse is not a consequence of a thermal diffusion process, since the phonons reach the equlibrium temperature in our single crystal very quickly. This can be seen from the heat capacity measurements above 0.5 K or in high magnetic field, where there is no slow relaxation process. In this case, the strong exponential time dependence is observed after 10-20 ms and yields the equilibrium heat capacity.

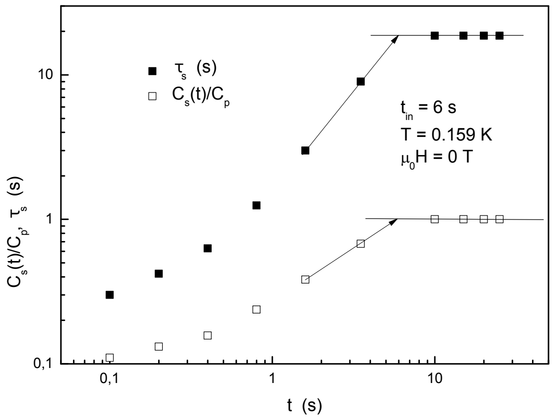

FIG. 2. White squares show the ratio of the short-time heat capacity C_s to the equilibrium heat capacity C_p as function of time for the data of Fig. 1. The ratio is constant at $t > t_{in} = 6$ s. $C_s(0.1\text{s})$ is about 9 times smaller the equilibrium heat capacity. The relaxation time becomes constant for $t > t_{in} = 6$ s. Black squares show the relaxation time $\tau_s(t)$ as a function of time determined from the data of Fig. 1. The relaxation time becomes constant for $t > t_{in} = 6$ s. $\tau_s(0.1\text{ s})$ is 60 times smaller than the equilibrium relaxation time due to an inner relaxation process.

More detailed information about the spectrum of an inner relaxation process can be obtained by the relaxation time method (RTM). Here, the temperature increases from T_0 by a permanent power P_h and, after some waiting time $t_w > t_{in}$, reaches the equilibrium value T_1. The heat link is defined as $R_p = (T_1 - T_0)/(U_h I_h)$. Then the heating power is switched off at $t = 0$ and the temperature is measured as a function of time. At sufficient long times ($t > t_{in}$) the inner relaxation process can be neglected and the time dependence of the temperature difference ΔT corresponds to

$$\Delta T_{eq} = \Delta T_{eq}^0 \exp(-t/\tau_{eq}) \tag{8}$$

(see Fig. 3). The fitting parameter τ_{eq} yields the equilibrium heat capacity

$$C_p = \frac{\tau_{eq}}{R_{hl}}. \tag{9}$$

When the spectrum of inner relaxation time corresponds

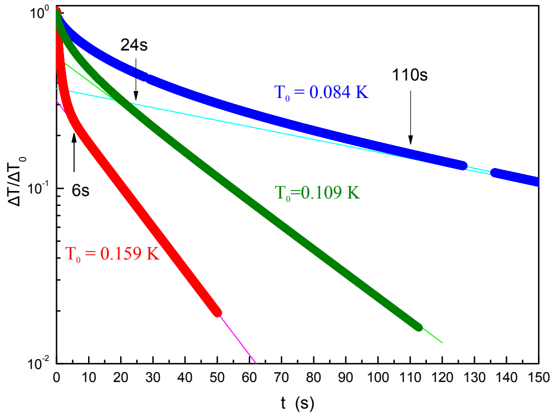

FIG. 3. The temperature difference $\Delta T/\Delta T_0 = (T - T_0)/(T_1 - T_0)$ as function of time after a constant heater power is switched off ($t = 0$) for different base temperatures $T_0 = 0.064$ K, 0.109 K and 0.159 K. The equilibrium heat capacity C_p can be obtained from the exponential law at $t > t_{in}$ according to Eq. (9) with t_{in} equals 110 s, 24 s and 6 s (arrows in the Figure), respectively. An inner relaxation process is observed for $t < t_{in}$. These data were used to determine the inner relaxation time spectrum.

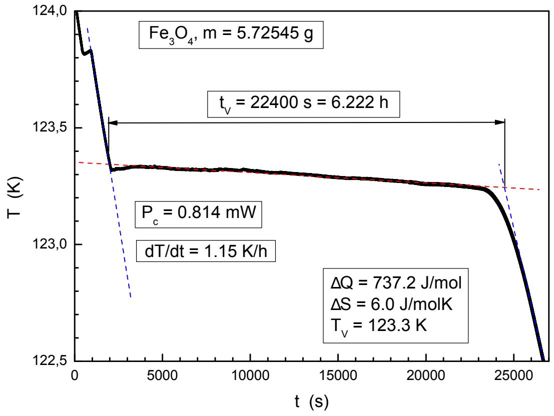

FIG. 4. The temperature as function of time around 123 K for a constant cooling power $P_c = 0.814$ mW. The temperature is about 6 h constant during the Verwey transition at 123.3 K (first order phase transition). The data allows to determine the latent heat $\Delta Q = 737.2$ J/mol and entropy difference $\Delta S = 6.0$ J/molK.

to a certain distribution with a finite width, the temperature difference at shorter time $t < t_{in}$ is well described by a *stretched exponential law.* The following model gives a good representation of the experimental data:

$$\Delta T = \Delta T_{eq} + \Delta T_{in}, \tag{10}$$

where ΔT_{eq} is defined by Eq. (8) and ΔT_{in} is given by a stretched exponential law

$$\Delta T_{in} = \Delta T_{in}^0 \exp(-t/\tau_m)^b \tag{11}$$

TABLE I. List of references (not exhaustive) where high values of the Verwey transition temperature T_V and entropy change (ΔS) have been reported.

Ref.	T_V (K)	ΔS (J/(mol K))
this work	123.3	6.0
Bland et al., 2009 [4]	120.4	5.8
Kim-Ngan et al., 2004 [5]	123.5	6.4
Shepherd et al., 1991 [6]	120.8 (aver.)	5.91 (aver.)
Paul, 2005 [7]	123.7	-
Masaaki et al., 1977 [8]	122.7	5.4+4.2
Gmelin et al., 1983 [9]	123.6	6.24

with constants ΔT_{in}, τ_m, and b being the fitting parameters.

II. THE VERWEY TRANSITION

Most of the experiments were carried out on cooling and several cooling rates were used: the *standard cooling* with a cooling rate of $dT/dt = 50$ K/h and a *slow cooling in a magnetic field of 1 T* in the temperature range between 130 K and 120 K with $dT/dt = 20$ K/h.

With standard cooling procedure the sample was cooled down together with the cryostat and the superconducting magnet using exchange gas in the sample chamber. With slow cooling procedure the sample was in vacuum during the whole cooling time. We monitored the temperature region of the Verwey transition in a special way. The sample temperature was kept constant at 130 K by regulation of the sample holder temperature. Then the temperature of the sample holder was reduced to 110 K while the temperature of the sample was kept at 130 K by the sample heater. Finally the sample heater was switched off and the sample temperature was measured (see Fig. 4). This procedure led to a cooling rate of $dT/dt = 1.15$ K/h.

Under these conditions we found that the Verwey transition dramatically slows the cooling of the sample for 22400 s at $T_V = 123.3$ K due to the heat coming out in the sample. Once the thermal conditions giving rise to this hiatus are exhausted the cooling of the sample resumes at the initial 1.15K/h rate. A zero drift was produced with the sample heater to determine the cooling power. In this way we can get the latent heat ΔQ and the entropy change ΔS at the Verwey transition

$$\Delta Q = I_h^2 R_h t_h M/m = 737 \text{ J/mol}, \quad (12)$$

$$\Delta S = \Delta Q/\Delta T_v = 6.0 \text{ J/(mol K)}, \quad (13)$$

where M is the molar mass. These results are in quite good agreement with earlier investigations of magnetite single crystal (see Table I).

The heat capacity around T_V was measured for increasing and decreasing temperatures of the sample holder. A giant anomaly of the heat capacity was observed at 122.9 K (see Fig. 5), 0.4K below its value determined in Fig. 4.

TABLE II. Results of the fitting with Eqs. (8), (10), and (11) of the relaxation time data.

T_0 (K)	$\Delta T_{in}^0/\Delta T_0$	τ_m (s)	b	$\Delta T_{eq}^0/\Delta T_0$	τ_{eq} (s)
0.159	0.71	0.95	0.83	0.29	18
0.109	0.46	4.5	0.67	0.54	32
0.084	0.62	16	0.7	0.38	120

The heat capacity increases from 70 J mol^{-1} K^{-1} at 122.2K to 24000 J mol^{-1} K^{-1} at 122.9K, nearly a factor of 400. The transition is very sharp and these measurements require a very small ΔT (0.1K) and a high stabilization of the sample temperature. The magnitude of the sharp anomaly at T_V, 400, has to be compared to that reported earlier[5], for what is believed a perfect stoichiometric synthetic crystal.

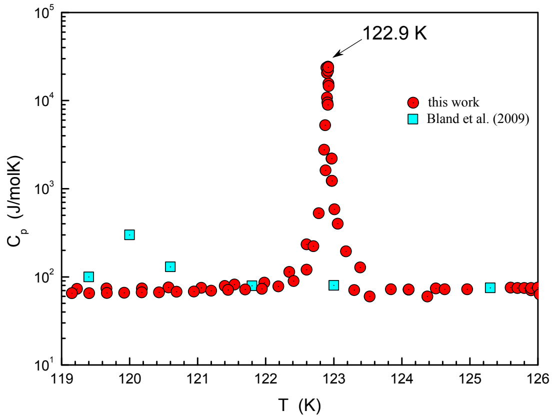

FIG. 5. Measured heat capacity near the Verwey transition using the pulse method. The maximum of the heat capacity occurs at 122.9 K, i.e. 0.4 K below its value in Fig. 4. The transition actually starts at 123.3 K, which we identify with T_V. Light blue squares correspond to the values from Ref. [4].

III. THERMAL RELAXATION DATA

Finally additional information can be extracted from the analysis of the thermal relaxation data. Eqs. (8), (10), and (11) yield a good agreement with the measured ΔT with $T_0 = 0.159$ K using the following parameters: $\Delta T_{eq}^0 = 0.0071$ K, $\Delta T_{in}^0 = 16.1$ K, $\tau_{eq} = 18$ s, $\tau_m = 0.95$ s, and $b = 0.78$. The corresponding parameters for all three temperatures are given in Table II.

Fig. 6 shows $\Delta T_{in}/\Delta T_0$ as a function of time for different three temperatures T_0. Arrows indicate the parameter t_{in} obtained in pulse experiments with the corresponding T_0.

The behavior of inner thermal relaxation may be described under the assumption that the relaxation times

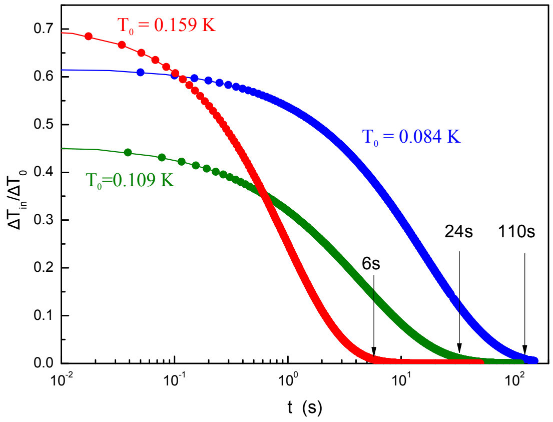

FIG. 6. The temperature difference ΔT_{in} caused by the inner relaxation process divided by the initial temperature difference ΔT_0 as function of time deduced from the data in Fig. 3. Marked in the figure are the different t_{in}. For more details see the text.

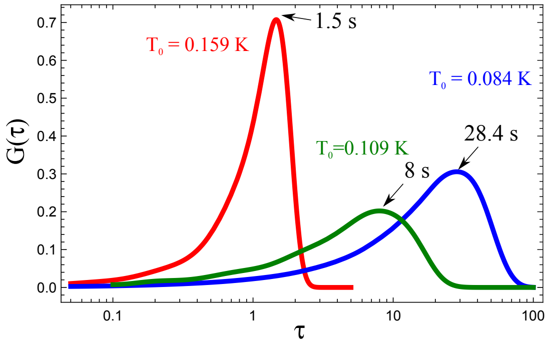

FIG. 7. The relaxation time spectrum $G(\tau)$ obtained from the data in Fig. 3. The maximum of the relaxation spectra are the τ_e.

have some finite-width distribution $G(\ln\tau)$

$$\frac{\Delta T_{in}(t)}{\Delta T_0} = \int_{\ln\tau_{min}}^{\infty} G(\ln\tau)\exp\left(-\frac{t}{\tau}\right) d\ln\tau. \qquad (14)$$

The relaxation time spectra obtained from the data shown in Fig. 6 are presented in Fig. 7. Interestingly t_{in} is of the same order of magnitude as the maximum of the relaxation time spectrum, τ_e, which stems from the fact that the width of the distribution function $G(\ln\tau)$ is almost unchanged for all three temperatures. Both characteristic times are well described by an Arrhenius law with the same activation energy of 0.5 eV (see Fig. 8). From this we conclude that the relaxation of two-level systems occurs through a thermoactivated mechanism, rather than through tunneling.

[*] emilio.lorenzo@neel.cnrs.fr
[†] katkov@theor.jinr.ru
[‡] osipov@theor.jinr.ru

[1] E. Nazarenko, J. E. Lorenzo, Y. Joly, J. L. Hodeau, D. Mannix, and C. Marin, Phys. Rev. Lett. **97**, 056403 (2006).
[2] J. E. Lorenzo, C. Mazzoli, N. Jaouen, C. Detlefs, D. Mannix, S. Grenier, Y. Joly, and C. Marin, Phys. Rev. Lett. **101**, 226401 (2008).
[3] O. Béthoux, R. Brusetti, J. Lasjaunias, and S. Sahling, Cryogenics **35**, 447 (1995).
[4] S. R. Bland, B. Detlefs, S. B. Wilkins, T. A. W. Beale, C. Mazzoli, Y. Joly, P. D. Hatton, J. E. Lorenzo, and V. A. M. Brabers, Journal of Physics: Condensed Matter **21**, 485601 (2009).
[5] N.-T. Kim-Ngan, W. Soszka, Z. Tarnawski, and A. Kozłowski, Physica B: Condensed Matter **353**, 164 (2004).
[6] J. P. Shepherd, J. W. Koenitzer, R. Aragón, J. Spal/ek, and J. M. Honig, Phys. Rev. B **43**, 8461 (1991).
[7] K. B. Paul, Central European Journal of Physics **3**, 115 (2005).
[8] M. Matsui, S. Todo, and S. Chikazumi, Journal of the Physical Society of Japan **42**, 1517 (1977).
[9] E. Gmelin, N. Lenge, and H. Kronmuller, physica status solidi (a) **79**, 465 (1983).

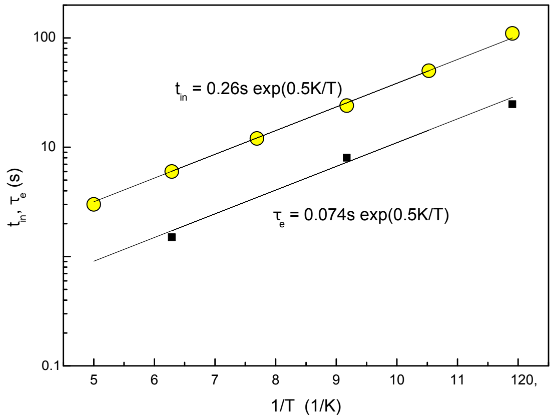

FIG. 8. The Arrhenius plot of the parameter t_{in} in zero magnetic field yields an activation energy $E_a/k_B = 0.5$ K. The same activation energy is obtained from the dependence of τ_e that corresponds to the maximum of the distribution functions in Fig. 7.
